# Multiplex Serology for Measurement of IgG Antibodies Against Eleven Infectious Diseases in a National Serosurvey: Haiti 2014–2015

**DOI:** 10.3389/fpubh.2022.897013

**Published:** 2022-06-09

**Authors:** YuYen Chan, Diana Martin, Kimberly E. Mace, Samuel E. Jean, Gillian Stresman, Chris Drakeley, Michelle A. Chang, Jean F. Lemoine, Venkatachalam Udhayakumar, Patrick J. Lammie, Jeffrey W. Priest, Eric William Rogier

**Affiliations:** ^1^The London School of Hygiene & Tropical Medicine, London, United Kingdom; ^2^Division of Parasitic Diseases and Malaria, Center for Global Health, Centers for Disease Control and Prevention, Atlanta, GA, United States; ^3^Population Services International/Organization Haïtienne de Marketing Social Pour la Santé, Port-au-Prince, Haiti; ^4^Programme National de Contrôle de la Malaria/MSPP, Port-au-Prince, Haiti; ^5^Division of Foodborne, Waterborne, and Environmental Diseases, National Center for Emerging and Zoonotic Infectious Diseases, Centers for Disease Control and Prevention, Atlanta, GA, United States

**Keywords:** multiplex assay, IgG detection, Haiti, integrated serosurveillance, infectious disease, seroprevalence

## Abstract

**Background:**

Integrated surveillance for multiple diseases can be an efficient use of resources and advantageous for national public health programs. Detection of IgG antibodies typically indicates previous exposure to a pathogen but can potentially also serve to assess active infection status. Serological multiplex bead assays have recently been developed to simultaneously evaluate exposure to multiple antigenic targets. Haiti is an island nation in the Caribbean region with multiple endemic infectious diseases, many of which have a paucity of data for population-level prevalence or exposure.

**Methods:**

A nationwide serosurvey occurred in Haiti from December 2014 to February 2015. Filter paper blood samples (*n* = 4,438) were collected from participants in 117 locations and assayed for IgG antibodies on a multiplex bead assay containing 15 different antigens from 11 pathogens: *Plasmodium falciparum, Toxoplasma gondii*, lymphatic filariasis roundworms, *Strongyloides stercoralis*, chikungunya virus, dengue virus, *Chlamydia trachomatis, Treponema pallidum*, enterotoxigenic *Escherichia coli, Entamoeba histolytica*, and *Cryptosporidium parvum*.

**Results:**

Different proportions of the Haiti study population were IgG seropositive to the different targets, with antigens from *T. gondii, C. parvum*, dengue virus, chikungunya virus, and *C. trachomatis* showing the highest rates of seroprevalence. Antibody responses to *T. pallidum* and lymphatic filariasis were the lowest, with <5% of all samples IgG seropositive to antigens from these pathogens. Clear trends of increasing seropositivity and IgG levels with age were seen for all antigens except those from chikungunya virus and *E. histolytica*. Parametric models were able to estimate the rate of seroconversion and IgG acquisition per year for residents of Haiti.

**Conclusions:**

Multiplex serological assays can provide a wealth of information about population exposure to different infectious diseases. This current Haitian study included IgG targets for arboviral, parasitic, and bacterial infectious diseases representing multiple different modes of host transmission. Some of these infectious diseases had a paucity or complete absence of published serological studies in Haiti. Clear trends of disease burden with respect to age and location in Haiti can be used by national programs and partners for follow-up studies, resource allocation, and intervention planning.

## Introduction

Tropical and other infectious diseases cause high morbidity and mortality worldwide, and many are co-endemic due to socioeconomic, environmental, climatological, and other factors (1). Epidemiology, control, and potential elimination of these diseases benefits from continued surveillance and monitoring for acute infection or past exposure. As symptomatic surveillance alone may not be a reliable indicator of infection for many tropical diseases, serological confirmation provides an effective way of estimating pathogen exposure within a population (2–6). Additionally, as infectious disease transmission is reduced in an area, standard diagnostic methods for many pathogens tend to provide less accurate estimates of true prevalence (7, 8). Serological assays that detect antibodies against pathogen-specific antigens are used for a variety of purposes such as providing history of infection of diseases within a population (9, 10), understanding transmission patterns (5), strategizing control and elimination efforts (11, 12), and assessing host immune status (13).

Conventionally, single-analyte detection methods such as Western blotting, lateral flow assays (LFAs), or enzyme-linked immunosorbent assays (ELISAs) have been used to detect human antibodies against infectious disease antigens. The bead-based multiplex platform for detecting and quantitating antibodies against multiple antigens is efficient for the concurrent analysis of an individual's serological profile to numerous infectious diseases (2, 9, 13, 14). Additional benefits include the time and reduced costs of multiplexing targets for several pathogens compared to traditional single-plex assays while remaining relatively easy to operate in a laboratory setting (15, 16). Thus, multiplex assays can offer a practical and more comprehensive understanding of epidemiologic patterns and co-endemic burdens of infectious diseases in an area (13, 17).

In this current study, a multiplex bead assay (MBA) was utilized to assess IgG antibody levels for 4,438 blood samples collected during a Haitian national community-based household survey that took place from December 2014 to February 2015. The MBA panel included 15 antigenic targets to evaluate exposure to 11 infectious diseases in the nation of Haiti. Data are displayed to estimate department-level and national-level seroprevalence estimates and trends by age categories.

## Materials and Methods

### Ethical Approvals and Sample Collection

The study protocol was approved by the Haitian Ministry of Health. Participant consent (and parental assent if under 15 years) was verbal. The Haitian population was sampled from December 2014 to February 2015 as part of the Global Fund grant against malaria (Round 8) implemented by Population Services International (PSI) Haiti as Principal Recipient. Enumeration areas throughout the country (*sections d*'é*numération*, SDE) were chosen on a proportional sampling by predicted malaria risk strata as determined by predictive modeling (18). A target of 20 households were randomly selected by field teams within each SDE, and all members of the household were offered the opportunity to participate. Blood was collected by fingerprick on Whatman 903 Protein Saver cards (GE Healthcare, Chicago, IL), dried overnight, and individually stored in plastic bags with desiccant at −20° C until shipment to the Centers for Disease Control and Prevention in Atlanta, GA, USA. Samples were assigned unique identification numbers that were not traceable to the individual. A total of 4,535 persons were enrolled in the survey, of which 4,438 (97.9%) provided DBS for serological assays. Participants in the survey were aged 1–99 years, with a median number of 30 persons sampled per SDE and 117 total SDEs sampled throughout the country. For the Haiti tracking results continuously (TRaC) survey, the study protocol was approved by the Haitian Ministry of Health and approved as a non-research activity by the Center for Global Health Human Research Protection Office (HRPO), US Centers for Disease Control and Prevention (CDC; Center for Global Health determination #2015-04).

Samples from U.S. resident blood donors were used to represent a population of persons putatively seronegative to tropical diseases not endemic to the U.S. All blood samples were from consenting adults who had screened negative for HIV and hepatitis B viruses and had no reported history of international travel in the last 6 months, and use was approved by CDC's Center for Global Health Institutional Review Board under non-engagement in human subjects research status.

### Antigens Used for Multiplex Bead Assay (MBA)

The 19-kDa fragment of the *P. falciparum* merozoite surface protein 1 (PfMSP1-19) was cloned from *P. falciparum* isolate 3D7 and expressed as previously described (17, 19, 20). The SAG2A antigen from *T. gondii* was cloned from the RH strain and produced recombinantly as described previously (21–23). The production of *Brugia malayi* roundworm recombinant antigens Bm33 and Bm14 have been described previously (24–27). *Wuchereria bancrofti* antigen Wb123 was a kind gift from T. Nutman (National Institutes of Health, Bethesda, MD) (28). The *Strongyloides stercoralis* NIE antigen (Ss-NIE-1) produced by L3 parasites was recombinantly produced as described previously (29, 30). The chikungunya virus envelope glycoprotein E1 was purchased through CTK Biotech (Porway, CA). The dengue virus serotype 2 virus-like particle was grown and isolated from transfected eukaryotic cell culture as described previously (31). The *Chlamydia trachomatis* antigens Pgp3 and CT694 were recombinantly expressed and purified as described previously (32). The recombinant *Treponema pallidum* antigen rp17 was purchased by Chembio Diagnostic Systems (Medford, NY) and recombinant TmpA through ViroGen (Boston, MA) and dialyzed overnight before bead coupling as described previously (2). Recombinant enterotoxigenic *E. coli* heat-labile enterotoxin B subunit (ETEC LTB) produced in *Pichia pastoris* was purchased from Sigma Aldrich (St. Louis, MO) (33). The *Entamoeba histolytica* LecA recombinant antigen was kindly provided by William Petri, Jr. (University of Virginia, Charlottesville, VA) and Joel Herbein (TechLab, Blacksburg, VA) (34, 35). The recombinant 27-kDa antigen from *Cryptosporidium parvum* (Cp23) has been previously described (36, 37). The antigen MBA panel is outlined in Table 1 and Supplementary Table 1.

**Table 1:** Infectious Diseases Represented and Antigens used for Multiplex Serology.

**Pathogen**	**Disease**	**Antigen**
*Plasmodium falciparum*	Malaria	PfMSP1-19
*Toxoplasma gondii*	Toxoplasmosis	Sag2A
*Wuchereria bancrofti*	Lymphatic filariasis	Wb123
*Brugia malayi*	Lymphatic filariasis	Bm14
*Brugia malayi*	Lymphatic filariasis	Bm33
*Strongyloides stercoalis*	Strongyloidiasis	NIE
Chikungunya virus (CHIKV)	Chikungunya	Chik E1
Dengue Virus Type 2 (DENV2)	Dengue	Dengue 2 VLP
*Chlamydia* spp.	Trachoma / Chlamydia	Pgp3
*Chlalamydia trachomatis*	Trachoma / Chlamydia	CT694
*Treponema* spp.	Yaws / Syphilis	rp17
*Treponema pallidum*	Yaws / Syphilis	TmpA
Enterotoxic *E. coli*	Diarrhea	ETEC-LTB
*Entamoeba histolytica*	Amoebiasis	LecA
*Cryptosporidium parvum*	Cryptosporidiosis	Cp23

### Antigen Binding to Beads

Antigens were covalently bound to polystyrene BioPlex® COOH beads (BioRad, Hercules, CA; 1715060XX) or Luminex® SeroMap beads (Luminex Corp, Austin, TX, L100-S0XX) by the commonly used EDC/Sulfo-NHS intermediate reaction. Previous comparisons between magnetic and non-magnetic beads have found comparable serological results between the two bead types (38–40). Reactive esters were formed on the carboxylated beads in the presence of 5 mg/mL EDAC (1-Ethyl-3-(3′-dimethylaminopropyl)carbodiimide) (EMD Millipore; 341,006) and 5 mg/mL Sulfo-NHS (N-hydroxysulfosuccinimide, ThermoScientific; 24,510) under light agitation for 20 min. Carboxyl to primary amine crosslinking took place in buffer at pH 5 (0.85% NaCl and 0.05 M 2-(N-morpholino)ethanesulfonic acid, MES) or at pH 7.2 (phosphate buffered saline, PBS, 10 mM PO_4_ and 0.85% NaCl) under light agitation for 2 h. Nonspecific protein binding was blocked by BSA incubation (PBS pH 7.2, + 1% bovine serum albumin, BSA) for 30 min, and beads were resuspended in blocking buffer with the addition of 0.02% NaN_3_ and protease inhibitors as described previously (25). Each antigen had been previously optimized to the appropriate coupling concentration and pH: CHIK-E1 (pH 5, 17 μg/mL); Dengue 2 VLP (pH 7.2, 30 μg/mL); *Brugia malayi* Bm14 (pH 7.2, 120 μg /mL); *Wuchereria bancrofti* Wb123 (pH 7.2, 120 μg/mL); Bm33 (pH 6.0 with 2M urea, 20 μg/mL); Enterotoxigenic *E. coli* (ETEC) heat-labile enterotoxin beta subunit (pH 5, 30 μg/mL); *Chlamydia trachomatis* Pgp3 pCT03 (pH 7.2, 120 μg/mL); *C. trachomatis* CT694 (pH 7.2, 30 μg/mL); *Treponema pallidum* TmpA (pH 5, 15 μg/mL); *T. pallidum* rp17 (pH 5, 15 μg /mL); *Toxoplasma gondii* SAG2A (pH 5, 12.5 μg/mL); *Plasmodium falciparum* MSP1 (pH 5, 30 μg/mL); *Strongyloides stercoralis* NIE (pH 7.2 with 2M urea, 20 μg/mL); *Cryptosporidium parvum* Cp23 (pH 5, 12.5 μg/mL); *Entamoeba histolytica* LecA (pH 5.0, 30 ug/mL). As an internal control to test for non-specific binding or any serum IgG against *Schistosoma japonicum* glutathione-*S*-transferase (GST) fused to recombinant antigens (41), a bead was included in the panel that was coupled to GST (coupling concentration of 15 μg/mL at pH5).

### Blood Spot Elution and MBA

A 6 mm circular punch corresponding to approximately 10 μL whole blood was taken from the center of each blood spot for elution. Samples were shaken in 100 μL protein elution buffer overnight at room temperature (PBS pH 7.2, 0.05% Tween-20, 0.05% NaN3) and stored at 4°C until further processing. Elution from blood spots provided an initial 1:10 dilution. Samples were further diluted 1:40 in Luminex sample diluent [PBS, 0.5% Polyvinyl alcohol (Sigma), 0.8% Polyvinylpyrrolidone (Sigma), 0.1% casein (ThermoFisher, Waltham, MA), 0.5% BSA (Millipore, Burlington, MA), 0.3% Tween-20, 0.02% NaN3, and 3 μg/mL *E. coli* extract to prevent non-specific binding] for a final whole blood dilution of 1:400, corresponding to a serum dilution of approximately 1:800 with the assumption of 50% hematocrit in whole blood. This serum dilution in the range of serum dilution previously utilized by our group and found to be able to provide accurate seroestimates for all infectious disease antigens on our multiplex panel.

For the MBA, a mix was prepared for all bead regions in 5 mL reagent diluent (PBS, 0.05% Tween20, 0.5% BSA, 0.02% NaN_3_). Filter bottom plates (Multiscreen 1.2 μm, Millipore) were pre-wetted with PBS-T, 50 μL bead mix (approximately 1,500 beads/analyte) added to wells and wells washed twice, and beads incubated with the sample in duplicate for 1.5 h under gentle shaking. Secondary antibodies tagged with biotin (1:500 monoclonal mouse anti-human total IgG (Southern Biotech); 1:625 monoclonal mouse anti-human IgG_4_ (Southern Biotech) were incubated with the beads for 45 min, and subsequent incubation with streptavidin-phycoerythrin (1:200, Invitrogen) for 30 min. Plates had a final wash incubation with reagent diluent for 30 min and were read on a Bio-Plex 200 machine to generate the median fluorescence intensity (MFI) signal for 50 beads/analyte. Background (bg) MFI was generated from blank wells containing only sample diluent, and this value was subtracted from each antigen's raw MFI to give an MFI-bg. The mean of MFI-bg values from duplicate wells was used for analysis, though previous studies from our group and others have also shown high reproducibility for MBAs when only singlet assay wells are run (42). Due to limited volumes of antigen-coupled beads, not all samples had data collected for IgG against all antigens. Total number of persons with IgG antibody data collected for each antigen is summarized in Supplementary Table 1.

### Determining Seropositivity Thresholds

Determining the MFI-bg assay signal threshold above which an individual was determined to be IgG positive (seropositive) for each of the antigens in the study was accomplished through different approaches. The MFI-bg signal thresholds are all shown in Supplementary Table 1. No cutoff estimate was available for Enterotoxigenic *E. coli* LT B subunit antigen as a negative population was not available for comparison (43).

#### Non-exposed U.S. Residents Approach

For all infectious diseases assayed in this study that were endemic only to tropical areas, the antigen panel for those diseases was assayed with blood samples from 92 U.S. residents who were unlikely to have been exposed to these infectious diseases. From this population of U.S. residents, the lognormal mean MFI signal plus three standard deviations were exponentiated to derive the seropositivity signal threshold (in MFI-bg units). This approach was used for the malaria (PfMSP1-19), lymphatic filariasis (Bm14, Bm33, Wb123), stronglyloides (NIE), chikungunya virus (E1), dengue virus (VLP), and *E. histolytica* (LecA) antigens. Histograms for MFI-bg signal distribution for all antigens included in this study for the Haitian study population vs. the US resident sample set are shown in Supplemental Figure 1.

#### Mixture Model Approach

Some pathogens included in this analysis are endemic in the US, and individuals cannot be assumed to be seronegative. To determine seropositive and seronegative subpopulations in a dataset, a 2-component mixture model strategy was used (Supplementary Figure 2). From the first distribution (component) of log-transformed data, which is assumed to be the distribution of the signal of the putative seronegative population, the mean plus three standard deviations were exponentiated to derive the seropositivity signal threshold (in MFI-bg units). This approach was used for the *T. gondii* (SAG2A), *C. trachomatis* (Pgp3, CT694), and *T. pallidum* (rp17, TmpA) antigens.

#### Receiver Operator Characteristic Curve Analysis

For responses to the *C. parvum* Cp23 antigen, the typical approach is to use a panel of Western blot positives and negatives to establish a cutoff by Receiver operator characteristic curve analysis (44). The beads used in this study were previously determined to have a cutoff of 1,870 MFI-bg by this method (45). However, since this study used only 50% of the serum concentration in each assay as the previous work, the cutoff was adjusted to 935 MFI-bg to account for the difference.

### Statistical Analysis

Statistical procedures were performed in SAS® 9.4 software (SAS Institute, Cary, NC), at the 5% significance level (alpha: 0.05), applying both Anderson-Darling and Cramér-von Mises null hypotheses. Descriptive statistics and histograms in SAS software were summarized with corresponding 95% confidence interval, using the PROC FREQ, PROC UNIVARIATE, and PROC MEANS statements. Ages were categorized into eight mutually exclusive groups (0–4 years, 5–10 years, 11–15 years, 16–20 years, 21–30 years, 31–40 years, 41–50 years, and >50 years) due to observed differences between antibody concentrations of younger and older populations. unweighted, two-component finite mixture models (FMM) of log-transformed data were compared using the FMM procedure in SAS. Logistic and linear regressions were created using PROC REG and PROC GLM. Analysis of potential correlation between antigens was produced through PROC LOGIT, and PROC CORR statements. Seroprevalence estimates were not generated for the enterotoxigenic *E. coli* LT B subunit antigen as exposure in the population is ubiquitous (43).

## Results

**Figure 1:**
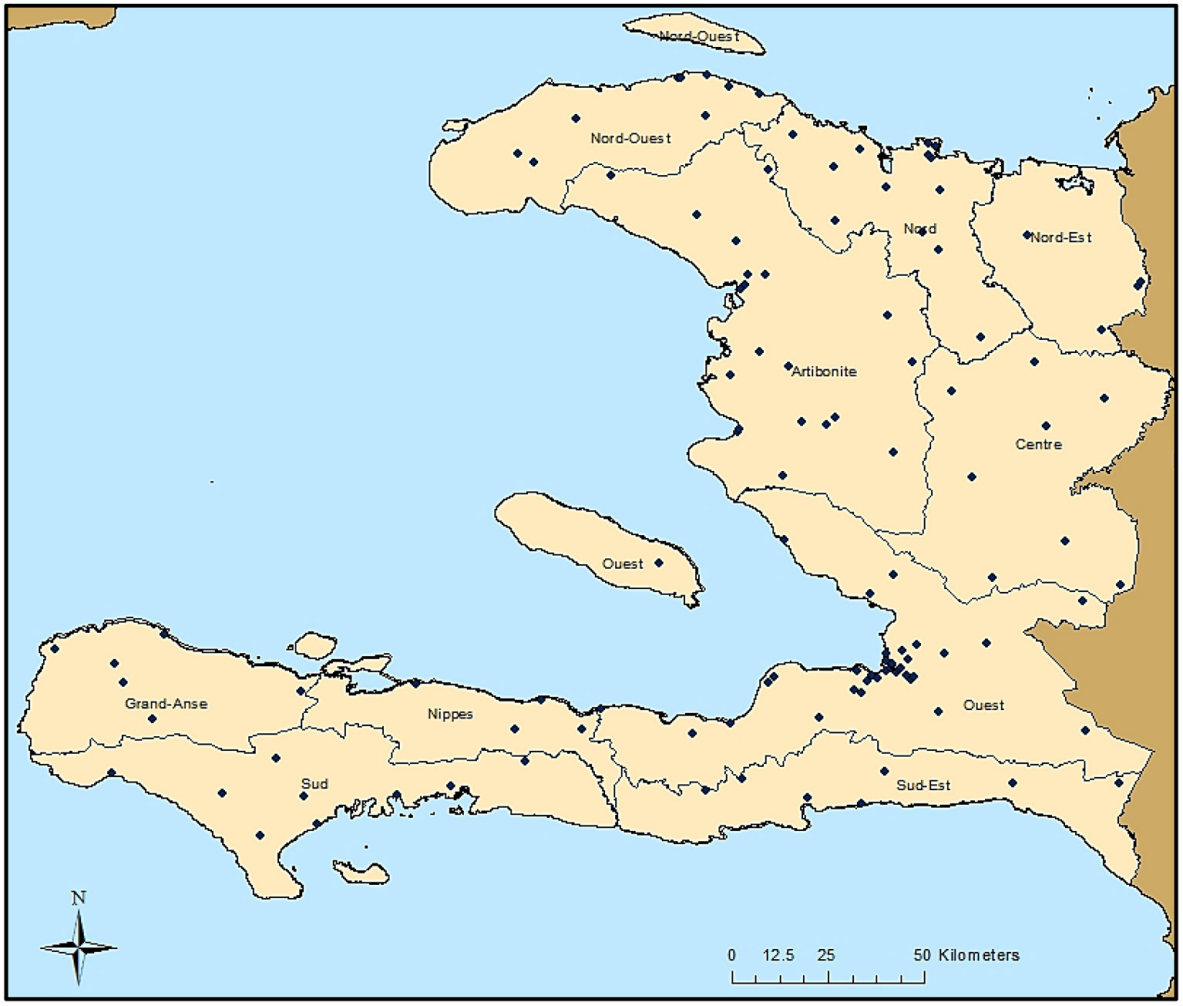
Sampling Locations in Haiti from the 2014/2015 Nationwide Survey. Each of the 117 sampling locations are indicated by a black dot. Boundaries of the ten Haitian departments are also shown.

As part of the 2014/2015 TRaC survey, 117 communities were sampled throughout Haiti, as shown in Figure 1. Though the sampling design was powered to present nationwide malaria estimates to gauge the relative disparities in seroprevalence among three different malaria risk strata, IgG seropositivity to different antigens is displayed by Haitian departments in Supplementary Table 2. For the Ouest department, estimates for the city of Port-au-Prince were displayed separately from the more rural areas, as this is a broad urban area that is densely populated. A large percentage of the population in all departments was seropositive to dengue virus serotype 2 (dengue 2 VLP) and chikungunya (Chik E1), ranging from 65.0–91.9% and 20.8–59.3%, respectively. Seropositivity to antigens for the parasitic pathogens *C. parvum* (Cp23) and *T. gondii* (SAG2A) was found to be high as well, with 26.1 and 45.0% of all persons seropositive, respectively. Among the lymphatic filariasis antigens, Wb123 and Bm14 never exceeded 3.6% seroprevalence for all persons within a department, whereas Bm33 ranged from 5.0–9.5% seroprevalence. Seropositivity to *P. falciparum* for the whole study population was 21.8% and ranged from 12.0% in urban Port-au-Prince to 37.1% in the rural Center department. The two antigens for *C. trachomatis* (Pgp3 and CT694) provided similar seropositivity estimates for the whole study population (41.7% vs. 35.2% respectively), as well as the two antigens for *T. pallidum* (rp17: 6.6% vs. TmpA: 5.0%).

**Figure 2:**
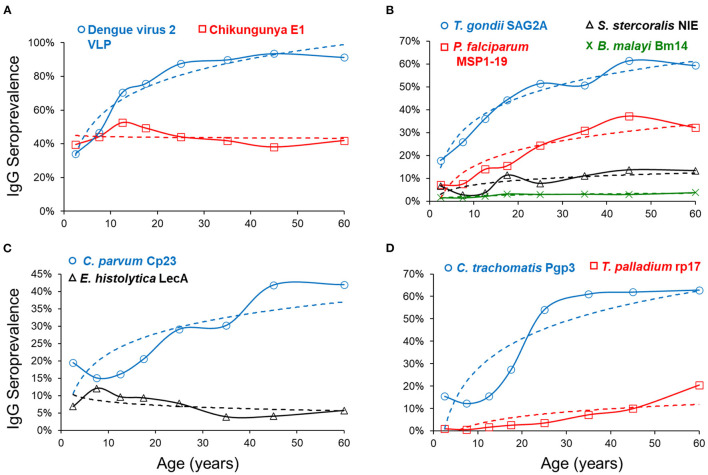
Seroprevalence by Age and Regression Fitting for Selected Antigens. By age category, mean seroprevalence (as plotted by percent IgG positive) was plotted on y-axis and age on x-axis. Dashed regression lines were fitted to a logarithmic equation for positivity by age and grouped into similar categories of arboviruses **(A)**, non-waterborne parasites **(B)**, waterborne pathogens **(C)**, and other bacterial pathogens **(D)**.

Figure 2 depicts mean seroprevalence to a subset of antigens by age, grouped into categories of disease or pathogen similarities. In Figure 2, single antigens were included to represent *W. bancrofti, C. trachomatis*, and *T. pallidum*. Seropositivity data were fitted to a logarithmic equation with intercept and slope estimates for all antigens (Supplementary Table 3). Positive slope estimates were highest for the dengue virus, *T. gondii*, and the *C. trachomatis* antigens. Only two antigens provided negative slopes (chik E1 and *E. histolytica* LecA), both of which were non-significant. When modeling for seropositivity by age, logarithmic regression provided strong goodness of fit (R^2^>0.75) for PfMSP1-19, SAG2A, Wb123, Bm14, dengue 2 VLP, pgp3, and Ct694 (Supplementary Table 3).

**Figure 3:**
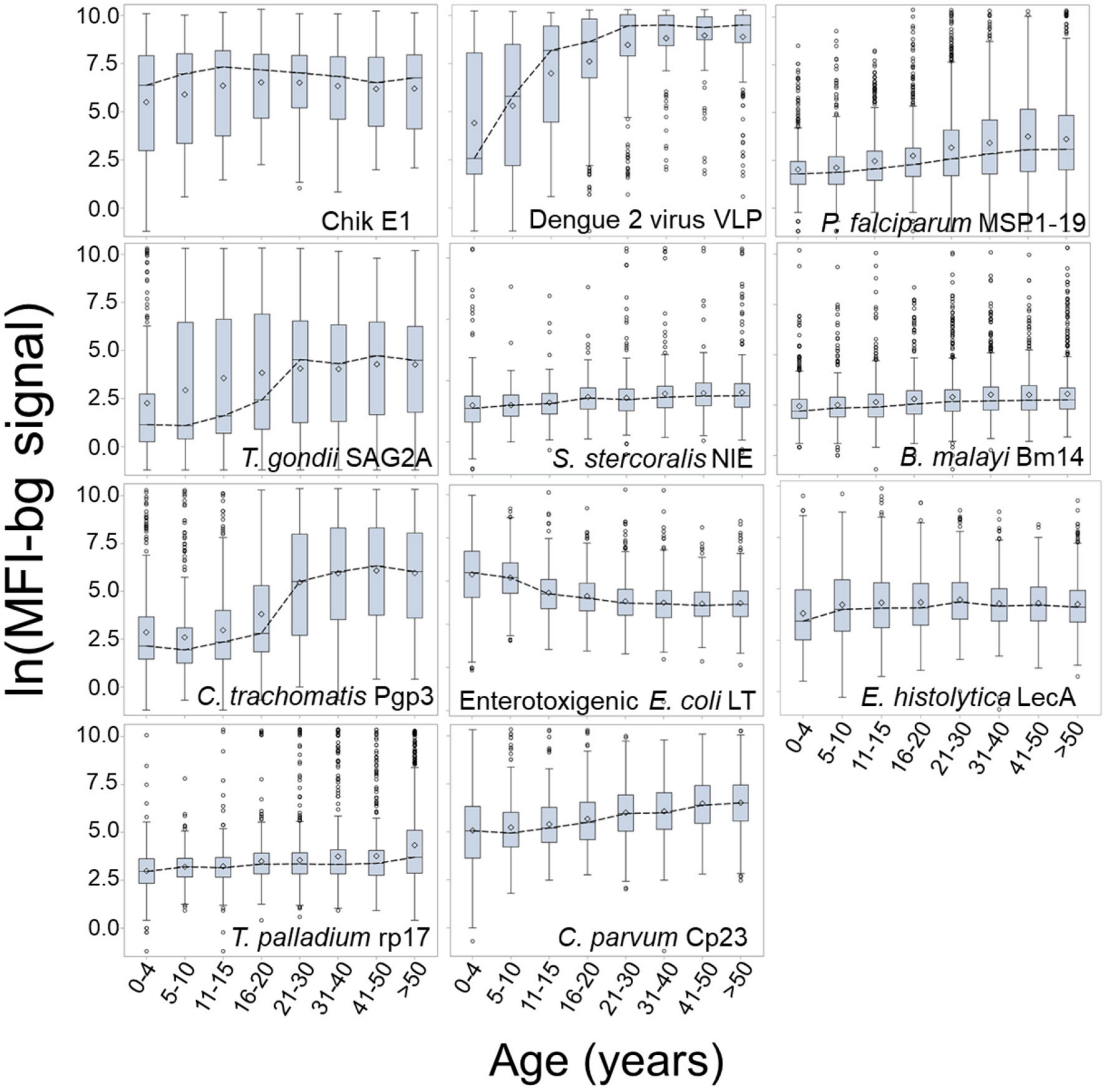
Dynamics in MFI-bg IgG Signal by Age for Selected Antigens. Data shown as boxplots for log-transformed median fluorescence intensity minus background (MFI-bg) assay signal for selected antigens by age category. Boxplots are displayed as interquartile rage (IQR) with whiskers extending 1.5x above and below IQR and circles for observations beyond this. A dashed line connects boxplot medians.

Figure 3 presents the log-transformed MFI-bg by age category for the same selected antigens in Figure 2 with the addition of ETEC-LTB, and regression estimates for the effect of age on IgG titer are shown in Supplementary Table 4. When modeling for acquisition or loss of MFI-bg assay signal by age, most estimates for the age parameter were found to be statistically significant within the regression model (Supplementary Table 4), with only the Wb123, Bm33, and chik E1 antigens not significant. Though the *C. parvum* Cp23 showed clear increases in seropositivity with age, the other antigen from a waterborne pathogen (*E. histolytica* LecA) showed a consistent negative slope when modeling for seropositivity (Supplementary Table 3) or MFI-bg signal (Supplementary Table 4) by age. All other antigens had consistent positive slopes for seropositivity and IgG acquisition with age except SAG2A (positive slope for seropositivity by age, negative slope for MFI-bg IgG response by age) and chik E1 (negative slope for seropositivity with age, positive slope for MFI-bg IgG intensity by age). Slopes for seropositivity and antibody acquisition by age were positively correlated (Supplementary Figure 3). For the ETEC-LT and dengue 2 VLP antigens, regression estimates were also generated for only young children to show IgG response's rapid loss (or gain) throughout their first years of life (Supplementary Table 4). The correlation of MFI-bg signal among all antigens is shown in Supplementary Table 5. Specifically, antigens from the same pathogens showed the highest correlation values: Pgp3 and Ct694 (rho = 0.83), rp17 and TmpA (rho = 0.64), Bm14, and Wb123 (rho = 0.60).

## Discussion

In this report, we show the capacity of the MBA to investigate exposure to multiple diseases of interest from samples gathered from December 2014 to February 2015 during a nationwide malaria survey in Haiti. Analyses took into account participants' ages and areas of residence, but future studies with demographic and spatial data could allow for more informative epidemiological outputs (46, 47). Haitian estimates for population-level exposure to each of the pathogens will be described below as grouped by infectious disease category and include examples of how serological data generated by MBA can be applied.

### Arboviruses

This serosurvey represented two arboviruses: chikungunya and dengue virus serotype 2. Our study found the transmission dynamics for these two arboviruses to be quite different, with the dengue virus antigen providing a population seroprevalence curve indicating increased likelihood of lifetime exposure as persons aged. Beginning immediately after birth, seroprevalence and IgG levels rise rapidly in the first 15 years of life. By age 30, Haitians had a >80% chance of being exposed to the dengue virus and typically displayed very high IgG titers. In contrast, the seroprevalence curve by age categories for chikungunya was mostly flat, likely indicative of the recent introduction of the disease into the country in 2014 and the rapid spread of this arbovirus among all age groups (48).

### Waterborne Pathogens

Antigens for two waterborne pathogens are included in this survey: *C. parvum* and Enterotoxigenic *Escherichia coli* (ETEC), both of which are important causes of childhood diarrhea (49). We observed small but consistent increases in antibody levels to the Cp23 antigen with age, similar to what was previously seen in Haiti (35). We found the IgG levels against the ETEC-LT antigen to be high in the youngest ages; levels decrease during the first 15 years of life and remain low among older age groups. This finding may suggest immune tolerance to this *E. coli* antigen, as noted for another *E. coli* antigen, lipopolysaccharide (LPS) (50). In this study population, seropositivity to the *E. histolytica* LecA antigen peaked around 10 years of age, though IgG levels also slowly increased with persons' age. The use of this antigen to assess *E. histolytica* serology has been very limited but has shown clear increases in seroconversion during the first years of life (35), which mirrors exposure dynamics to this parasite in children (51, 52).

### Other Parasites

*Plasmodium falciparum* is transmitted through *Anopheles* spp. mosquitos and is the primary causative agent of malaria in Haiti (53, 54). Our current study showed a consistent increase in seropositivity, and the population antibody levels with age. Modeled PfMSP1-19 seropositivity estimated that a person aged 45 years would be 3.7-fold more likely to be seropositive than someone aged 5 years. Malaria serology data can be applied to understanding areas of ongoing transmission (17). The percentages of population seropositive to PfMSP1-19 were lowest in the Port au Prince metropolitan area (12.0%) and highest in the Center department (37.1%), consistent with known lower *P. falciparum* exposure in urban areas due to poor mosquito vector habitat (55).

Toxoplasmosis is a zoonotic infection caused by a single celled parasitic protozoan, *Toxoplasma gondii*. Transmission occurs when eating undercooked, contaminated meat or by oral ingestion of the oocyst stage when humans come into contact with infected cat feces (56). As *T. gondii* infection typically goes into latency in the human host (57), seropositivity indicates lifetime infection (58). Our study found reliable increases in IgG prevalence to the SAG2A antigen with age, indicating past and current stable transmission of this parasite. Our data estimated that by the time a Haitian reaches 25 years of age, the risk of exposure to *T. gondii* is approximately 50%. A previous cohort study in Leogane, Haiti, had estimated seroprevalence to *T. gondii* of 25% among children 0–12 years old, similar to the estimates of 22% presented in this study (21).

Adult lymphatic filariasis (LF) worms live in the lymph system, and microfilariae circulate in the blood, and this disease is found throughout the tropical and subtropical areas of the world (33, 59). In Haiti, LF is caused by the roundworm *Wucheria bancrofti*, and current targets for elimination will benefit from continual serosurveillance efforts as the endemic range is reduced (60). In this study, we employed three filarial antigens to identify seropositive persons in this low-transmission setting. A low proportion of the population was seropositive to the worm antigens, with low (but positive) slope estimates indicating an increase in seroprevalence and IgG titer with age, as has been seen in other low transmission settings (10).

The roundworm *Strongyloides stercoralis* is the causative agent of strongyloidiasis. This soil-transmitted helminth is transmitted when the skin comes into contact with free-living larvae in contaminated soil, and most people infected are asymptomatic (32, 61, 62). *Strongyloides* seroprevalence estimates have previously been proposed as a pragmatic tool for population exposure (41, 63), and our study found an overall low Haitian seroprevalence (<15% in any department) to the *S. stercoralis* NIE antigen with increasing seroprevalence by age. The lowest seroprevalence was found in Artibonite (4.6%), the highest in Grand'Anse (14.9%), and a nationwide seroprevalence of 9.2%. Surveillance for roundworms through serological data could be utilized for directing mass drug administration campaigns in areas where active infection and parasite prevalence are difficult to estimate.

*C. trachomatis* and *T. pallidum* are sexually transmitted infections (STIs, causing chlamydia, and syphilis, respectively) that also cause non-venereal infections in children. Ocular *C. trachomatis* infection can lead to trachoma, the world's leading infectious cause of blindness (33). Yaws, a skin infection that can lead to bone and soft tissue damage, is caused by *T. pallidum* spp *pertenue*. The childhood infection and STI are serologically indistinct for each of these pathogens. Our study found consistent increases in antibodies against *C. trachomatis* antigens Pgp3 and CT694 with age, with the most pronounced increases during the ages of sexual debut, likely indicative of STI exposure. Haiti is not thought to be endemic for trachoma, and these data do not suggest high trachoma transmission intensity (11, 64, 65).

Antibodies to the treponemal antigen rp17 represent historical exposure, whereas TmpA-specific antibodies represent recent infection (66). Only 0.52% of under 15–year-olds were antibody-positive to both antigens. No data on yaws are available from Haiti, while the sampling frame may not be granular enough to detect pockets of yaws transmission, these data are suggestive of little or no yaws transmission in the surveyed areas. An interesting contrast in seroprevalence of *C. trachomatis* and *T. pallidum* was seen in the adult population, with approximately 60% of the >20–year-olds seropositive to chlamydial antigens and <10% seropositive to antigens indicative of syphilis.

Limitations to this study include that laboratory-based serological assays depend on the sensitivity and specificity of the assays used for IgG detection. Some diseases do not have well-defined antigens that are known to elicit strong IgG responses or have antigens with known IgG cross-reactivity issues with responses from other pathogens. Additionally, some infectious diseases (such as those infecting the respiratory or gastrointestinal tract) are known to elicit strong IgA responses, so the measurement of only IgG in this study may have reduced the sensitivity of seropositivity measurement. As this study only used one serum dilution, for IgG quantification purposes against each individual antigen, different serum dilutions for each target would be most optimal. Seropositivity cut-off values need to be evaluated by each group employing these antigen targets, especially in elimination programs where populations have decreased exposure and finding active infections is difficult (10, 17). Increasing survey sample sizes can help overcome statistically biased estimates and increase precision. Defining seroconversion, boosting effects after re-exposure, antibody decay, and immunocompetency of the host are all primary concerns for some infectious diseases. Continued investigation is required to correctly interpret serology data for different diseases. The seropositivity cutoff for Cp23 was determined 1:800 serum dilution, whereas this was previously at done at a 1:400 dilution (35). This may have overestimated the true cutoff value and resulted in lower-than-expected seroprevalence values. Among the factors listed above, another limitation to this study is that the sampling design was powered for the modeled malaria active infection prevalence in Haiti (18), but the cluster design, enrollment in households, and attempt at nationwide representativeness can still provide insight into population exposure to other infectious diseases. Additionally, the survey was cross-sectional, and regression estimates of data representing trends over time assume consistent dynamics of endemicities and transmission intensity. Future studies in Haiti should investigate if similar findings would be observed.

This nationwide Haiti survey for malaria provided an opportunity to employ a 15-antigen MBA panel measuring IgG presence and titer to 11 infectious diseases. As some pathogens are cleared from the host within a few days or weeks, assaying for antibodies greatly augments the window of time in which to survey for exposure in a population. In addition, accurate surveillance for recurrent-type infectious diseases can be confounded by asymptomatic infections, poor access to healthcare or healthcare reporting, or poor diagnostics. Understanding the co-endemic disease burden on a national level allows for collaborative strategies of multiple stakeholders focused on combined interventions at the community level. Multiple programs, especially those targeting multiple diseases, can be monitored simultaneously through one well-designed, population-representative integrated survey (41, 60, 67). A follow-up cross-sectional survey throughout Haiti with the same sampling design could prove valuable for monitoring changes in seroestimates in the Haitian population since this 2014–2015 survey.

## Data Availability Statement

The raw data supporting the conclusions of this article will be made available by the authors, without undue reservation.

## Ethics Statement

The studies involving human participants were reviewed and approved by Haitian Ministry of Health; Center for Global Health Human Research Protection Office (HRPO), US Centers for Disease Control and Prevention. Written informed consent to participate in this study was provided by the participants' legal guardian/next of kin.

## Author Contributions

SJ and JL facilitated field studies. MC, VU, PL, JP, and ER conceptualized the experiments. DM, PL, and JP provided laboratory supplies. ER collected data. YC and ER performed data analyses and drafted the manuscript. DM, KM, GS, CD, MC, and JP provided technical expertise. All authors reviewed and approved the manuscript for submission.

## Funding

Global Fund to Fight AIDS, Tuberculosis and Malaria funded the Haiti nationwide survey. Funds for publication are from the Centers for Disease Control and Prevention.

## Author Disclaimer

Use of trade names is for identification only and does not imply endorsement by the Public Health Service or by the U.S. Department of Health and Human Services. The findings and conclusions in this report are those of the author(s) and do not necessarily represent the official position of the Centers for Disease Control and Prevention.

## Conflict of Interest

The authors declare that the research was conducted in the absence of any commercial or financial relationships that could be construed as a potential conflict of interest.

## Publisher's Note

All claims expressed in this article are solely those of the authors and do not necessarily represent those of their affiliated organizations, or those of the publisher, the editors and the reviewers. Any product that may be evaluated in this article, or claim that may be made by its manufacturer, is not guaranteed or endorsed by the publisher.
