# Supplementary material for: Multiplex Serology for Measurement of IgG Antibodies Against Eleven Infectious Diseases in a National Serosurvey: Haiti 2014–2015
Source: Front Public Health. 2022 Jun 9;10:897013. doi: 10.3389/fpubh.2022.897013 (PMC9218545; doi:10.3389/fpubh.2022.897013)
Supplement: Supplementary file 1 [file Data_Sheet_1.PDF]

## Supplementary Material

### Supplementary Table 1. MBA assay antigen formats and seropositivity cutoff values

| Antigen | Format | MFI-bg Threshold for Seropositivity | Number of Samples With Data Collected (% of 4,438) |
|---|---|---|---|
| PfMSP1-19 | Recombinant | 61 | 4424 (99.7%) |
| SAG2A | Recombinant | 40 | 1682 (37.9%) |
| Wb123 | Recombinant | 411 | 4395 (99.0%) |
| Bm14 | Recombinant | 530 | 4438 (100.0%) |
| Bm33 | Recombinant | 457 | 4309 (97.1%) |
| NIE | Recombinant | 493 | 2238 (50.4%) |
| Chik E1 | Recombinant | 1353 | 4438 (100.0%) |
| Dengue 2 VLP | Virus-like particle | 543 | 1253 (28.2%) |
| Pgp3 | Recombinant | 161 | 3325 (74.9%) |
| CT694 | Recombinant | 74 | 4118 (92.8%) |
| rp17 | Recombinant | 314 | 4395 (99.0%) |
| TmpA | Recombinant | 46 | 4395 (99.0%) |
| ETEC-LT | Recombinant | NA | 4411 (99.4%) |
| LecA | Recombinant | 190 | 2833 (63.4%) |
| Cp23 | Recombinant | 925 | 2790 (62.9%) |

**Supplemental Table 2. Seropositivity to infectious disease antigens by Haitian department**

| Department | PfMSP1-19 | SAG2A | Wb123 | Bm14 | Bm33 | NIE | Chik E1 | Dengue 2 VLP | Pgp3 | CT694 | rp17 | TmpA | LecA | Cp23 |
|---|---|---|---|---|---|---|---|---|---|---|---|---|---|---|
| Port-au-Prince | 12.0 | 54.3 | 1.0 | 3.1 | 5.5 | 9.1 | 59.3 | 87.3 | 38.7 | 35.1 | 4.1 | 4.1 | 8.2 | 28.4 |
| Artibonite | 24.9 | 30.8 | 1.3 | 3.5 | 8.3 | 4.6 | 46.4 | 68.8 | 41.9 | 40.7 | 6.2 | 5.6 | 7.5 | 28.0 |
| Centre | 37.1 | 37.9 | 0.4 | 0.7 | 6.7 | 12.6 | 21.6 | 67.2 | 46.5 | 35.3 | 6.0 | 3.2 | 10.3 | 29.0 |
| Grand'Anse | 16.0 | 56.7 | 0.0 | 3.2 | 5.0 | 14.9 | 25.9 | 91.9 | 55.2 | 39.6 | 8.6 | 5.5 | 2.9 | 41.0 |
| Nippes | 35.9 | 54.6 | 0.0 | 1.4 | 6.2 | 9.1 | 45.5 | ND | 28.3 | 27.6 | 11.0 | 7.6 | 4.7 | 27.1 |
| Nord | 20.2 | 58.8 | 2.4 | 3.6 | 9.5 | 11.4 | 35.4 | 74.2 | 48.2 | 35.6 | 7.6 | 5.4 | 10.3 | 18.2 |
| Nord'Est | 19.5 | 50.0 | 0.0 | 0.0 | 8.4 | 11.8 | 20.8 | 65.0 | 37.5 | 37.7 | 5.8 | 2.6 | 5.8 | 26.0 |
| Nord'Ouest | 20.8 | 24.8 | 2.3 | 3.5 | 9.2 | 6.2 | 42.8 | ND | 51.1 | 37.1 | 6.0 | 4.0 | 7.1 | 28.3 |
| Ouest (Outside PAP) | 21.8 | 43.6 | 2.6 | 2.6 | 7.2 | 9.8 | 47.4 | 75.3 | 36.2 | 28.1 | 7.4 | 6.1 | 3.2 | 26.6 |
| Sud'Est | 20.1 | 48.4 | 0.5 | 2.2 | 7.1 | 9.0 | 38.4 | 79.8 | 45.1 | 35.7 | 10.3 | 5.8 | 5.8 | 33.3 |
| Sud | 30.1 | 37.5 | 0.3 | 2.7 | 5.3 | 7.5 | 45.3 | 90.0 | 35.6 | 35.8 | 6.5 | 5.0 | 8.2 | 34.6 |
| **Nationwide** | **21.8** | **45.0** | **1.3** | **2.8** | **7.2** | **9.2** | **43.5** | **75.6** | **41.7** | **35.2** | **6.6** | **5.0** | **7.2** | **26.1** |

**Supplementary Table 3. Modeling seropositivity by age**

| Antigen | Intercept | Slope | R square |
|---|---|---|---|
| PfMSP1-19 | -0.081 | 0.102 | 0.84 |
| SAG2A | 0.013 | 0.146 | 0.95 |
| Wb123 | 0.007 | 0.007 | 0.79 |
| Bm14 | 0.007 | 0.007 | 0.79 |
| Bm33 | 0.072 | 0.001 | 0.00 |
| NIE | 0.029 | 0.004 | 0.53 |
| Chik E1 | 0.454 | -0.005 | 0.01 |
| Dengue 2 VLP | 0.142 | 0.207 | 0.94 |
| Pgp3 | -0.169 | 0.194 | 0.76 |
| CT694 | -0.146 | 0.166 | 0.82 |
| rp17 | -0.081 | 0.049 | 0.58 |
| TmpA | -0.040 | 0.030 | 0.72 |
| LecA | 0.116 | -0.015 | 0.29 |
| Cp23 | 0.028 | 0.083 | 0.65 |

**Supplementary Table 4. Change in antibody MFI-bg levels to different antigens with age**

| Antigen | Intercept, βo (95% CI) | Age, βx (95% CI) | Chi-Square Statistic | Model p value |
|---|---|---|---|---|
| PfMSP1-19 | 108 (-28 , 244) | 13 (9, 17) | 45.3 | **< 0.0001** |
| SAG2A | 2098 (1713, 2482) | -21 (-31, -10) | 14.9 | **0.0001** |
| Wb123 | 37 (20, 54) | 0.3 (-0.2, 0.7) | 1.22 | 0.268 |
| Bm14 | 78 (16, 139) | 2 (0, 3) | 4.01 | **0.045** |
| Bm33 | 203 (156, 250) | 0.1 (-1, 1) | 0.05 | 0.827 |
| NIE | 270 (110, 429) | 7 (3, 12) | 10.6 | **0.001** |
| Chik E1 | 2114 (1927, 2280) | 1 (-4, 6) | 0.14 | 0.705 |
| Dengue 2 VLP | 5763 (4899, 6628) | 145 (122, 168) | 150.2 | **< 0.0001** |
| Pgp3 | 1089 (708, 1471) | 58 (47, 68) | 111.9 | **< 0.0001** |
| CT694 | 268 (143, 394) | 13 (9, 16) | 51.8 | **< 0.0001** |
| rp17 | -62 (-245, 120) | 25 (20, 30) | 91.3 | **< 0.0001** |
| TmpA | 20 (4, 36) | 0.5 (0, 1) | 3.86 | **0.045** |
| ETEC-LT | 953 (880, 1025) | -14 (-16, -12) | 195.2 | **< 0.0001** |
| LecA | 84 (73, 94) | -0.4 (-0.7, -0.1) | 8.7 | **0.003** |
| Cp23 | 926 (711, 1140) | 13 (7, 19) | 20.0 | **< 0.0001** |
| | | | | |
| **ETEC 0-10y olds** | 2161 (1864, 2457) | -159 (-207, -110) | 40.9 | **< 0.0001** |
| **DENGV2 0-15y olds** | 1594 (136, 3052) | 377 (206, 547) | 18.8 | **< 0.0001** |

| | PfMSP1-19 | Chik E1 | Dengue 2 VLP | ETEC-LT | SAG2A | Wb123 | Bm14 | Bm33 | Pgp3 | CT694 | rp17 | TmpA | NIE | LecA | Cp23 |
|---|---|---|---|---|---|---|---|---|---|---|---|---|---|---|---|
| **PfMSP1-19** | 1 | 0.09202 | 0.29118 | -0.0527 | 0.15599 | 0.3015 | 0.37721 | 0.2715 | 0.33254 | 0.33498 | 0.34405 | 0.36243 | 0.33128 | 0.28561 | 0.24035 |
| | | <.0001 | <.0001 | 0.0005 | <.0001 | <.0001 | <.0001 | <.0001 | <.0001 | <.0001 | <.0001 | <.0001 | <.0001 | <.0001 | <.0001 |
| | 4424 | 4424 | 1248 | 4424 | 1677 | 4381 | 4424 | 4296 | 3314 | 4105 | 4381 | 4381 | 2231 | 2826 | 2783 |
| **Chik E1** | | 1 | 0.35254 | -0.00514 | 0.1619 | 0.18853 | 0.20327 | 0.11099 | 0.08343 | 0.12715 | 0.18925 | 0.16348 | 0.15059 | 0.08872 | 0.1277 |
| | | | <.0001 | 0.7322 | <.0001 | <.0001 | <.0001 | <.0001 | <.0001 | <.0001 | <.0001 | <.0001 | <.0001 | <.0001 | <.0001 |
| | | 4438 | 1253 | 4438 | 1682 | 4395 | 4438 | 4309 | 3325 | 4118 | 4395 | 4395 | 2238 | 2833 | 2790 |
| **Dengue 2 VLP** | | | 1 | -0.13824 | 0.20922 | 0.26575 | 0.28936 | 0.24509 | 0.36493 | 0.33291 | 0.24493 | 0.26356 | 0.278 | 0.12554 | 0.19409 |
| | | | | <.0001 | <.0001 | <.0001 | <.0001 | <.0001 | <.0001 | <.0001 | <.0001 | <.0001 | <.0001 | <.0001 | <.0001 |
| | | | 1253 | 1253 | 1253 | 1210 | 1253 | 1210 | 1210 | 1253 | 1253 | 1210 | 1167 | 1253 | 1210 |
| **ETEC-LT** | | | | 1 | -0.1041 | 0.084 | 0.05713 | 0.03083 | -0.1791 | -0.11064 | 0.04429 | 0.0568 | 0.04693 | 0.174 | -0.029 |
| | | | | | <.0001 | <.0001 | 0.0001 | 0.043 | <.0001 | <.0001 | 0.0033 | 0.0002 | 0.0264 | <.0001 | 0.1258 |
| | | | | 4438 | 1682 | 4395 | 4438 | 4309 | 3325 | 4118 | 4395 | 4395 | 2238 | 2833 | 2790 |
| **SAG2A** | | | | | 1 | 0.20695 | 0.21477 | 0.17233 | 0.21134 | 0.19312 | 0.20626 | 0.1671 | 0.20932 | 0.1328 | 0.19874 |
| | | | | | | <.0001 | <.0001 | <.0001 | <.0001 | <.0001 | <.0001 | <.0001 | <.0001 | <.0001 | <.0001 |
| | | | | | 1682 | 1639 | 1682 | 1639 | 1639 | 1682 | 1639 | 1639 | 1596 | 1682 | 1639 |
| **Wb123** | | | | | | 1 | 0.59604 | 0.40162 | 0.2065 | 0.283 | 0.41905 | 0.48001 | 0.47277 | 0.30085 | 0.22447 |
| | | | | | | | <.0001 | <.0001 | <.0001 | <.0001 | <.0001 | <.0001 | <.0001 | <.0001 | <.0001 |
| | | | | | | 4395 | 4395 | 4309 | 3282 | 4075 | 4352 | 4352 | 2238 | 2790 | 2747 |
| **Bm14** | | | | | | | 1 | 0.45584 | 0.3018 | 0.36638 | 0.47392 | 0.53873 | 0.44399 | 0.39075 | 0.23337 |
| | | | | | | | | <.0001 | <.0001 | <.0001 | <.0001 | <.0001 | <.0001 | <.0001 | <.0001 |
| | | | | | | | 4438 | 4309 | 3325 | 4118 | 4395 | 4395 | 2238 | 2833 | 2790 |
| **Bm33** | | | | | | | | 1 | 0.20076 | 0.23079 | 0.29275 | 0.32108 | 0.37202 | 0.31021 | 0.15625 |
| | | | | | | | | | <.0001 | <.0001 | <.0001 | <.0001 | <.0001 | <.0001 | <.0001 |
| | | | | | | | | 4309 | 3196 | 3989 | 4266 | 4266 | 2152 | 2704 | 2661 |
| **Pgp3** | | | | | | | | | 1 | 0.83458 | 0.29519 | 0.26712 | 0.25964 | 0.20881 | 0.30246 |
| | | | | | | | | | | <.0001 | <.0001 | <.0001 | <.0001 | <.0001 | <.0001 |
| | | | | | | | | | 3325 | 3091 | 3282 | 3325 | 2024 | 2152 | 2152 |
| **CT694** | | | | | | | | | | 1 | 0.35538 | 0.3518 | 0.28429 | 0.24062 | 0.29423 |
| | | | | | | | | | | | <.0001 | <.0001 | <.0001 | <.0001 | <.0001 |
| | | | | | | | | | | 4118 | 4075 | 4075 | 2238 | 2833 | 2790 |
| **rp17** | | | | | | | | | | | 1 | 0.63956 | 0.36378 | 0.31513 | 0.27057 |
| | | | | | | | | | | | | <.0001 | <.0001 | <.0001 | <.0001 |
| | | | | | | | | | | | 4395 | 4352 | 2195 | 2790 | 2747 |
| **TmpA** | | | | | | | | | | | | 1 | 0.50189 | 0.32787 | 0.28969 |

| | | | | | | | | | | | | | | | |
|---|---|---|---|---|---|---|---|---|---|---|---|---|---|---|---|
| | | | | | | | | | | | | | <.0001 | <.0001 | <.0001 |
| | | | | | | | | | | | | 4395 | 2195 | 2790 | 2790 |
| **NIE** | | | | | | | | | | | | | 1 | 0.2659 | 0.23936 |
| | | | | | | | | | | | | | | <.0001 | <.0001 |
| | | | | | | | | | | | | | 2238 | 2238 | 2195 |
| **LecA** | | | | | | | | | | | | | | 1 | 0.19323 |
| | | | | | | | | | | | | | | | <.0001 |
| | | | | | | | | | | | | | | 2833 | 2790 |
| **Cp23** | | | | | | | | | | | | | | | 1 |
| | | | | | | | | | | | | | | | |
| | | | | | | | | | | | | | | | 2790 |

**Supplemental Table 5. Spearman correlation coefficients, p values, and number observations of IgG Levels among antigen panel targets**

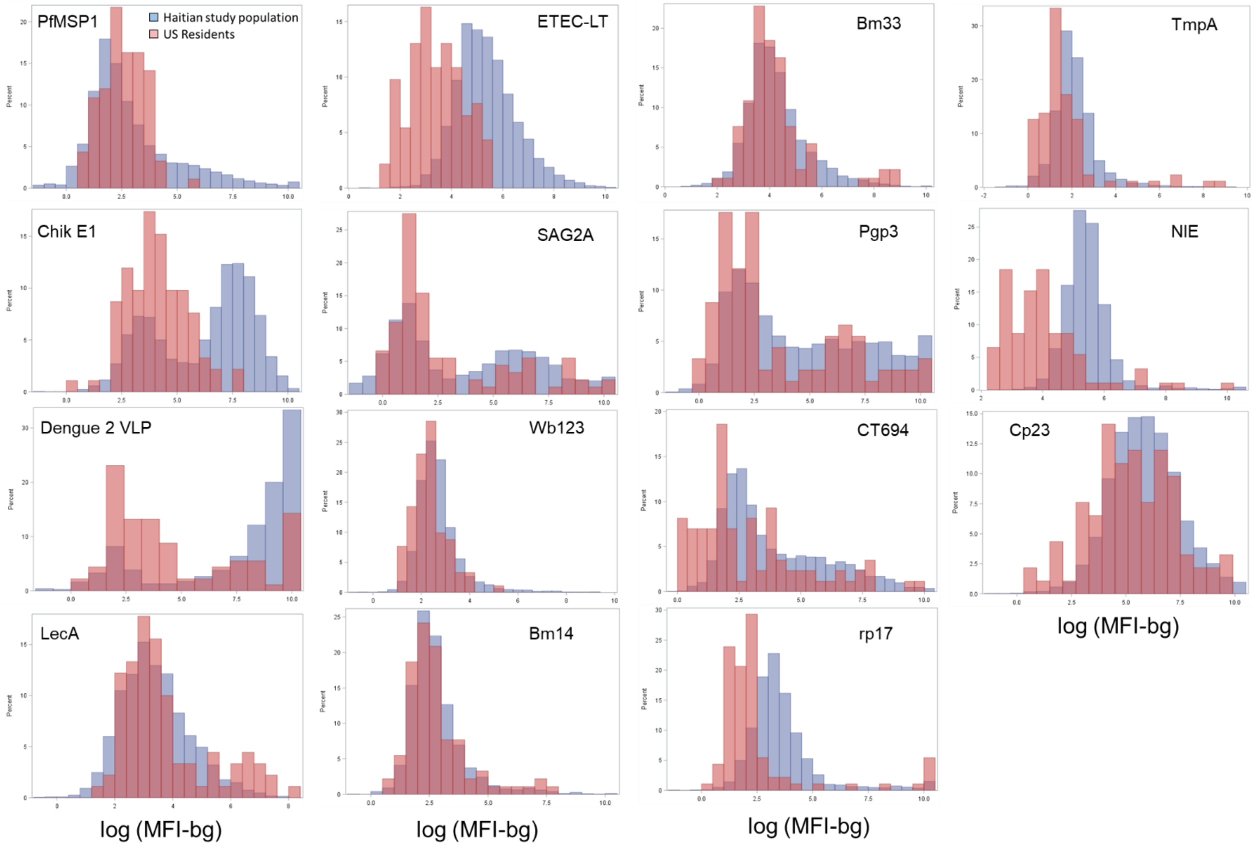


**Supplemental Figure 1. Histograms of log-transformed MFI-bg signal for all antigens utilized in this study for Haitian sample population and US resident population.** Histogram for Haitian study participants shown in blue and for US resident sample set shown in red.

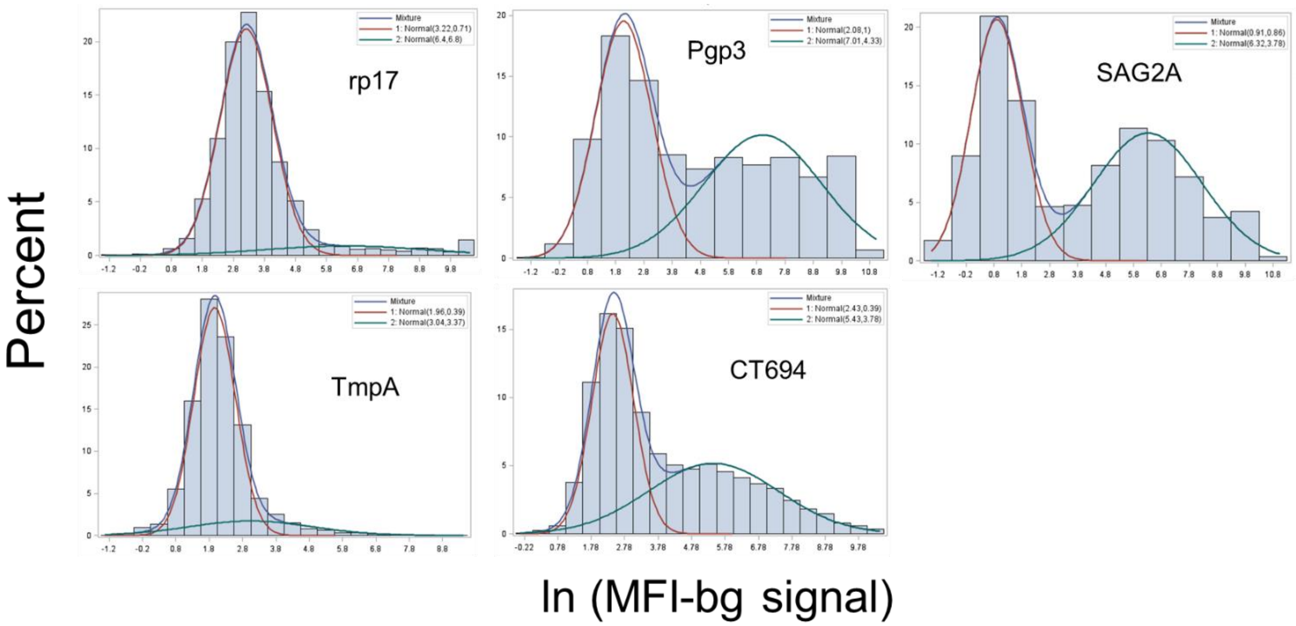


**Supplemental Figure 2. Use of the Finite Mixture Model to Derive Seropositivity Cutoff Threshold**. Log-transformed data from the Haiti survey was fitted to a two-component mixture model for antigens that U.S. residents have greater likelihood of exposure. For each antigen's figure legend, estimates of mean and variance are displayed for both components. Seropositivity threshold value for each antigen was calculated adding three standard deviations to the mean of the first component (as displayed by red line) and exponentiating back to linear scale.

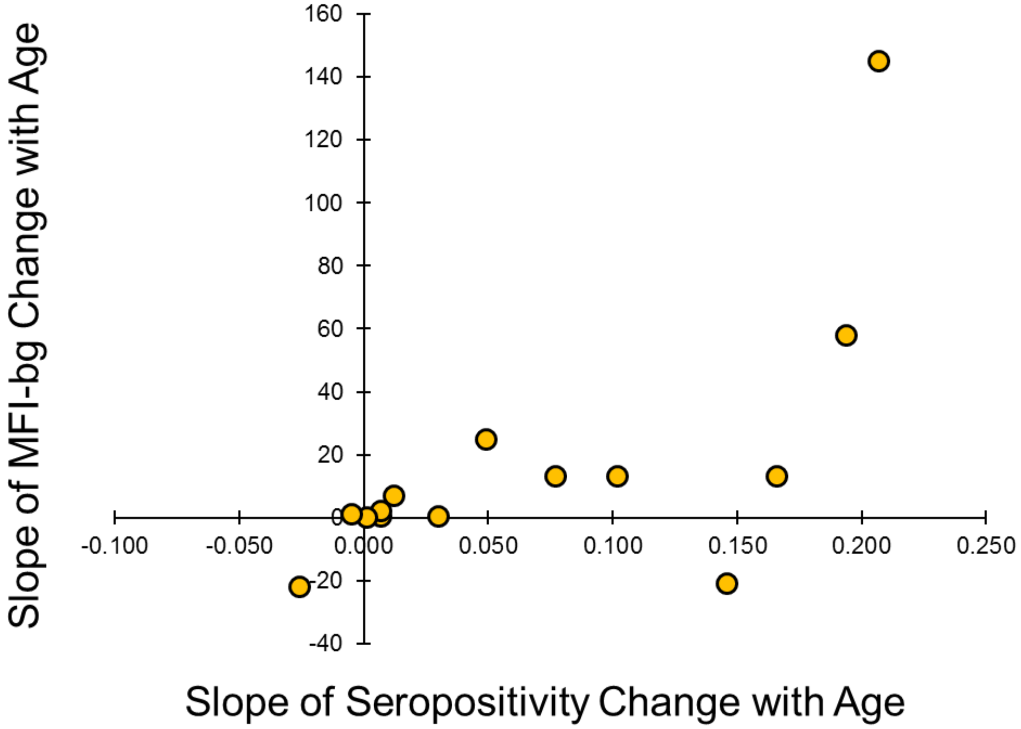


**Supplemental Figure 3. Relationship between regression slopes for seropositivity by age versus MFI-bg signal by age.** The slopes of the seropositivity regression in Fig 2/Supp Table 3 are displayed on the x-axis and the slopes of the change in MFI-bg with age in Fig 3/Supp Table 4 on the y-axis.
